# Unveiling the imprinted dance: how parental genomes orchestrate seed development and hybrid success

**DOI:** 10.3389/fpls.2024.1455685

**Published:** 2024-09-27

**Authors:** Muthusamy Muthusamy, Subramani Pandian, Eun-Kyuong Shin, Ho-Keun An, Soo-In Sohn

**Affiliations:** Biosafety Division, Department of Agricultural Biotechnology, National Institute of Agricultural Sciences, Rural Development Administration, Jeonju, Republic of Korea

**Keywords:** imprints, epigenetic regulators, ploidy dosage, hybridization barriers, hybrid vigor, seed size, parent-of-origin effect, RNA-directed DNA methylation

## Abstract

Parental epigenetic asymmetries, which contribute to the monoallelic expression of genes known as imprints, play a critical role in seed development in flowering plants. Primarily, differential DNA methylation patterns and histone modifications on parental alleles form the molecular basis of gene imprinting. Plants predominantly exhibit this non-Mendelian inheritance phenomenon in the endosperm and the early embryo of developing seeds. Imprinting is crucial for regulating nutrient allocation, maintaining seed development, resolving parental conflict, and facilitating evolutionary adaptation. Disruptions in imprinted gene expression, mediated by epigenetic regulators and parental ploidy levels, can lead to endosperm-based hybridization barriers and hybrid dysfunction, ultimately reducing genetic diversity in plant populations. Conversely, imprinting helps maintain genetic stability within plant populations. Imprinted genes likely influence seed development in various ways, including ensuring proper endosperm development, influencing seed dormancy, and regulating seed size. However, the functions of most imprinted genes, the evolutionary significance of imprinting, and the long-term consequences of imprinting disruptions on plant development and adaptation need further exploration. Thus, it is clear that research on imprinting has immense potential for improving our understanding of plant development and ultimately enhancing key agronomic traits. This review decodes the possible genetic and epigenetic regulatory factors underpinning genomic imprinting and their positive and negative consequences on seed development. This study also forecasts the potential implications of exploiting gene imprinting for crop improvement programs.

## 1 Introduction

Genomic imprinting reflects the epigenetic asymmetries that generate epigenetically unequal parental landscapes, contributing to parent-of-origin-dependent (monoallelic) expression of genes, which play critical roles in embryo and seed development in flowering plants (Adams et al., 2000; Pires et al., 2016; Städler et al., 2021; Pliota et al., 2024). This non-Mendelian phenomenon, where parent-of-origin dictates allele expression at the molecular level, leads to differential phenotypes and traits in offspring (Xu et al., 2014). Imprinting is the inevitable consequence of conflicting selective forces acting on differentially expressed parental alleles (Autran et al., 2005; Pliota et al., 2024). Genes expressed mostly from the maternal origin are known as Maternally Expressed Genes (MEGs) and those from the paternal origin as Paternally Expressed Genes (PEGs). Differences in the expression of parent-of-origin alleles are typically measured in reciprocal hybrids at heterozygous loci. The exact mechanisms by which imprinting evolved, manifests, and is maintained in plants are still being explored. Nonetheless, the research reports so far reveal that imprinting is crucial for mediating parent-offspring conflict, maintaining species boundaries, facilitating adaptation, and balancing genetic contributions (Ohto et al., 2005; Erilova et al., 2009; Pires, 2014; Rodrigues and Zilberman, 2015). To be precise, imprinting influences crucial developmental processes and traits, contributing to the evolutionary fitness and success of plant species. This underscores the biological and evolutionary significance of genomic imprinting. Therefore, understanding imprinting mechanisms and their consequences in plants will be of great importance for practical applications in agriculture and plant breeding.

The current understanding suggests that imprinting acts as an evolutionary mechanism to resolve the parental conflicts over resource provisioning, as a host defense mechanism against transposons and parasites in gametes, or as a consequence of natural selection for superior performance of offspring (Barlow, 1993; Yoder et al., 1997; Scott et al., 1998; Jiang and Kohler, 2012; Pires, 2014; Ashe et al., 2021; Sato and Köhler, 2022; Pliota et al., 2024). Deciphering the code of genomic imprinting is important to understand the mechanism by which parental genomes communicate with offspring through imprinting. Imprinting doesn’t involve direct communication but relies predominantly on DNA methylation/demethylation codes predetermined on genes that regulate gene expression in offspring, thereby regulating their phenotypes and other agronomic traits (Adams et al., 2000). The molecular mechanisms underlying this imprinted gene expression can involve different aspects of gene expression: promoter methylation, histone modifications, small interfering RNAs (siRNAs), and long non-coding RNAs (lncRNAs). Emerging studies have identified several epi-alleles and their regulators, like DNA methyl transferases, DNA demethylating DNA glycosylases, siRNAs, plant-specific RNA polymerase IV (Pol IV), and Polycomb group proteins, as contributing to the establishment and maintenance of gene imprinting predominantly in the endosperm of a developing seed (Adams et al., 2000; Wolff et al., 2011; Batista and Köhler, 2020; Grover et al., 2020; Satyaki and Gehring, 2022). Differential DNA methylation patterns and post-translational modifications of histones on the parental alleles form the molecular basis of gene imprinting in plants (Shirzadi et al., 2011; Waters et al., 2011; Xu et al., 2014). Of these, parent-specific DNA methylation/inheritance of epigenetic modifications have a strong association with genomic imprinting in plants. Following DNA methylation, the maternally expressed Polycomb Repressive Complex 2 (PRC2), which catalyzes H3K27me3, is found to be the second most imprinting regulatory mechanism in plants (Piskurewicz et al., 2016). *De novo* DNA methylation during gametogenesis imposes differential epigenetic modifications and sets the imprints for parent-of origin gene effects, while post-fertilization DNA methylation maintains the imprints (Autran et al., 2005). In particular, parental epigenetic asymmetries/imprints are added by means of methylation and demethylation during both female and male gametogenesis (Bauer and Fischer, 2011). DEMETER-mediated demethylation in the female gametophyte creates an asymmetry in methylation between parental copies (Gehring et al., 2009). The correct balance of these parental landscapes underlies imprinting and modulates gene expression, affecting seed development and mature seed size (Städler et al., 2021). Interestingly, these differentially methylated regions also known as epi-alleles, represent varying degrees of methylation patterns of loci between lines have been demonstrated to be stably inherited over many generations (Kooke et al., 2015; Botet and Keurentjes, 2020).

**Figure 1:**
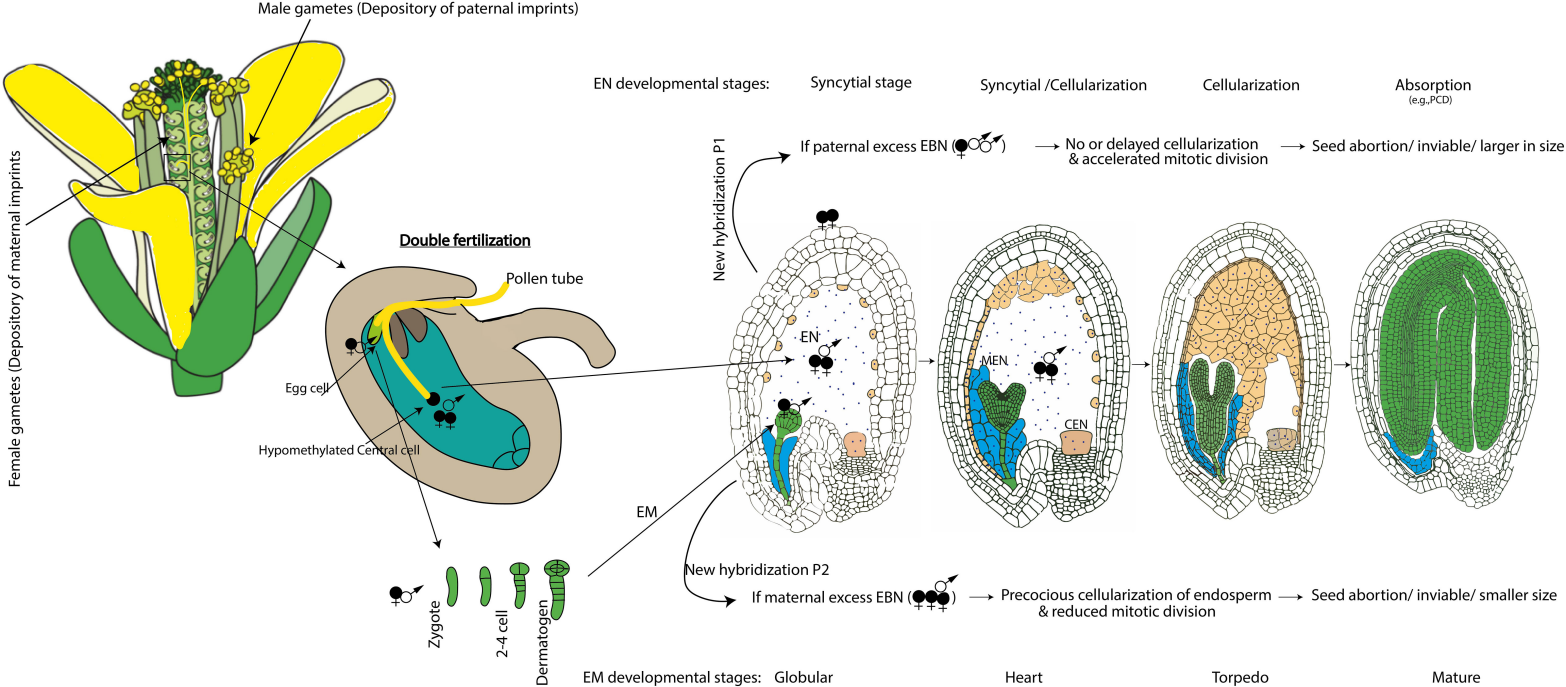
Schematic representation of inheritance pattern of parental imprints and their parent of origin-effect on endosperm development in the developing seed of Arabidopsis. EN, endosperm; MEN, micropylar endosperm; CEN, chalazal endosperm; EM, embryo; EBN, endosperm balance number; New hybridization possibilities P1 and P2, predominantly leading to abnormal EBN in some of the interspecific crosses or individual with different ploidy levels. Parental imprints set during gametogenesis and imprint maintenance after fertilization determines the endosperm development and early embryogenesis as part of seed development. Double fertilization involves two fertilization events within the ovule of a flower, leading to the formation of both the endosperm (sperm cell fused with central cell in the ratio of 2 maternal: 1 paternal genome composition) and embryo (sperm cell fused with egg cell in the ratio of 1 maternal and 1 paternal genome contribution), as part of coordinated seed development in flowering plants including Arabidopsis. EBN 2:1 ensures proper endosperm development while abnormal EBN can cause defective endosperm and ultimately affect the size and viability of the developing seed.

Plants exhibit imprinting predominantly in the endosperm, reflecting the differences in gamete epigenetic composition. Investigation of genomic DNA methylation distribution revealed relative hypomethylation in the endosperm, suggesting that endosperm tissues are crucial for imprinted gene expression (Xu et al., 2014). Interestingly, endosperm imprinted genes are found to be clustered. For instance, 109 of 297 imprinted genes were clustered on rapeseed chromosomes (Rong et al., 2021). Further, transposable elements were most enriched in both upstream and downstream of the imprinted genes, which is more than non-imprinted genes (Rong et al., 2021). Moreover, evidence shows that the degree of conservation of imprinted regions between species is relatively low. These regions are differentially regulated across species in response to growth stages, physiological needs, and environmental signals, which would be useful in plant acclimatization (Botet and Keurentjes, 2020). A study by Yuan et al. (2017) investigating the role of MEG and PEG in rice grain development revealed that one-third of MEGs and nearly one-half of PEGs were associated with grain yield quantitative trait loci. Additionally, imprinted genes affect the demand and supply of nutrients during plant endosperm development (Xu et al., 2014). The recent discovery of allelic dosage mediated by a siRNA pathway engaged in RNA-directed DNA methylation (RdDM) in endosperm has highlighted the role of non-coding RNAs in imprinting (Autran et al., 2005). In flowering plants, studies of parent-of-origin effects have mostly identified genes that are only transcribed from a maternally inherited allele. For instance, DEMETER, a plant DNA glycosylase responsible for DNA demethylation, activates the maternal MEDEA allele, which in turn controls seed development through the expression of PHERES1 (MADS-box gene) (Autran et al., 2005). Paternal-allele-specific expression of individual loci is also widely prevalent in the endosperm. Pires et al. (2016) demonstrated that reciprocal interactions between parental genomes can influence seed development, particularly in the context of the MEA gene in Arabidopsis. Hence, understanding the interplay between parental genomes can inform breeding strategies. In contrast to endosperm, a relatively small number of MEGs and PEGs have been reported to be imprinted in the embryo (Autran et al., 2005) (Montgomery and Berger, 2021). In 2009, MATERNALLY EXPRESSED IN EMBRYO 1 (*MEE1*) gene was identified as imprinted in both the embryo and endosperm (Jahnke and Scholten, 2009). In rice, triple knockout of the genes *BBM1*, *BBM2*, and *BBM3* causes embryo arrest and abortion, which are fully rescued by male-transmitted BBM1 (Khanday et al., 2019), suggesting that imprinted genes are crucial for embryogenesis just as they are for endosperm development. Genomic imprinting, by modulating the expression of maternally or paternally inherited alleles, is a predominant molecular basis of hybrid seed failure and hybrid vigor/heterosis in interploidy crosses (Lafon-Placette et al., 2018). Unequal gene expression patterns affecting maternally and paternally derived genomes during endosperm development play a major role in the maternal inheritance of seed dormancy (Piskurewicz et al., 2016; Sato and Köhler, 2022). The inheritance of imprinted genes can be seen as a way for parents to indirectly ‘provision’ their offspring with gene expression patterns that favor acclimatization to current environmental conditions (Ashe et al., 2021). Taken together, it is imperative that imprinting is crucial for seed development (**Figure 1**). These specific differentially methylated regions (DMRs) can therefore be considered epigenetic quantitative trait loci, offering potential for exploitation in crop improvement programs (Cortijo et al., 2014; Kooke et al., 2015).

## 2 Deciphering the code of genomic imprinting: DNA methylation and chromatin modification

Plants respond to fluctuating environmental cues and stress signals through epigenetic modifications central to gene regulation, phenotypic plasticity, development, and the preservation of genome integrity (Ashe et al., 2021). The epigenetic marks (e.g., DNA methylation), along with their full genetic components in seeds, can be inherited (Kooke et al., 2015), indicating that epigenetic modifications can have transgenerational effects (Quadrana and Colot, 2016; Liu et al., 2020). Specifically, DNA methylation is a conserved epigenetic modification affecting various processes including transcriptional gene silencing, regulation of transposal elements, parental imprinting, parent-of-origin effects, development, and seed viability. It functions by associating with histone modifications, chromatin remodeling, and influencing the accessibility of DNA to transcription factors (Adams et al., 2000; Xiao et al., 2006; Ashe et al., 2021). DNA methylation is found in the CG, CHG, and CHH sequence contexts (where H is A, C or T) in plants, and disruption of DNA methylation patterns can cause developmental and yield-related defects (Gallego-Bartolomé, 2020). There is an intricate interplay between DNA methylation and gene imprinting, whereby DNA methylation establishes asymmetric patterns during gametogenesis, leading to differential gene expression. The process of establishing methylation patterns in imprinted genes often involves the RNA-directed DNA methylation (RdDM) pathway. In plants, RdDM pathway is responsible for specific *de novo* methylation, often silencing gene expression at transcription levels. Wherein small interfering RNAs (siRNAs) can target imprinted control elements (ICEs), recruiting enzymes like Domains Rearranged Methyltransferase 2 (DRM2) to specific cytosines for methylation within or near the imprinted gene (He et al., 2011; Grover et al., 2020). ICEs are often composed of repetitive DNA sequences found flanking or internal to imprinted genes (MacDonald, 2012). While the exact mechanisms of imprinted gene regulation in plants are still under active investigation, ICEs are believed to play a crucial role, similar to their role in animals (Raissig et al., 2011). ICEs could act as promoters, enhancers, silencers, or locus control regions of target parent alleles, either by promoting or reducing the methylation and/or chromatin modifications processes (Raissig et al., 2011). It is interesting to note that the epigenetic state of ICEs controls the imprinted expression of all genes in one imprinted cluster (Koerner and Barlow, 2010). Similar to imprinted genes, ICEs are often species- and locus-specific. ICE elements could be involved in DNA methylation or histone modifications, ultimately leading to the silencing of one parental allele. However, unlike in animals where specific ICE motifs have been identified, plant ICEs lack a well-defined consensus sequence, making the identification of ICEs in plants is challenging to unravel their role in genomic imprinting (Gehring et al., 2011; Rodrigues and Zilberman, 2015) (Gehring et al., 2011). Methyltransferases, primarily Methyltransferase 1 (MET1), are responsible for maintaining established methylation pattern (Shirzadi et al., 2011). In model plant Arabidopsis, genes responsible for DNA methylation and demethylation have been identified as DNA methyltransferase MET1 and DME, respectively (Pires, 2014). Previous studies on Arabidopsis, caster bean (Xu et al., 2014), maize have demonstrated that the loss of maternal methylation in the central cell is expected to facilitate genomic/gene imprinting. This hypomethylation is possibly attributable to active DME in the central cell prior to fertilization (Sato and Köhler, 2022). As a result of DNA demethylation in the central cell, maternally expressed genes (MEGs) such as MEA, FWA, FIS2, and FIE2 were identified to be imprinted in the endosperm as reviewed by Pires et al (Pires, 2014).

In the context of gene imprinting, siRNAs can help establish and maintain the differential methylation patterns that distinguish parental alleles. They might target ICEs associated with imprinted genes and interact with the methylation code left on genes. Emerging studies have demonstrated that siRNAs play a significant role in regulating gene imprinting, particularly in the endosperm of seeds (Liu et al., 2020). Significant changes in siRNA profiles and their distribution patterns during gametogenesis are likely to reprogram gene expression towards seed development (Khanday and Sundaresan, 2021). Research suggests that a pathway involving NRPD1 is crucial for siRNA-mediated imprinting in Arabidopsis seeds (Kirkbride et al., 2019). Interestingly, only maternally derived siRNAs are detected in the endosperm, which possibly suggesting their maternal origin. Studies proven that siRNA involvement in RdDM are essential for early seed development (Xin et al., 2014; Grover et al., 2020) hence changes in siRNA profiles or expression levels can affect seed size (Xin et al., 2014). Further, the presence of highly expressed siRNAs in the seed coat and endosperm strengthens their involvement in seed development (Grover et al., 2020). Depending on the siRNA itself and its parental origin, it can target genes inherited from either parent, thus creating an asymmetric expression pattern characteristic of imprinted genes. Similarly, paternal Pol IV-dependent siRNAs (easiRNAs) regulate gene imprinting and dosage balance in the endosperm for the establishment of reproductive barriers in plants. Studies have indicated that Pol IV-derived siRNAs guide the RdDM machinery to target sites, establishing methylation patterns that are essential for the expression of imprinted genes and maintaining genomic stability (Zemach et al., 2013). This regulation is crucial for preventing transposon activation and ensuring normal seed development (Slotkin et al., 2009). Nonetheless, the field of siRNA-mediated imprinting in plants is still evolving, and the specific sRNAs and the precise mechanisms for targeting genes based on parental origin are largely unexplored, warranting further research to elucidate the biological significance of siRNAs in gene imprinting and seed development. At this juncture, it is clear that siRNAs are crucial for establishing proper DNA methylation patterns and gene imprinting in developing seeds.

Research reports also pointed out that chromatin factors like Polycomb group (PcG) proteins are important for establishment and maintenance of gene imprinting (Schuettengruber and Cavalli, 2009; Moreno-Romero et al., 2016). To be specific, the FIS- PRC2 (FERTILIZATION INDEPENDENT SEED-Polycomb Repressive Complex 2) complex, also referred to as the “molecular switch” establishes trimethylation marks on lysine 27 of histone H3 (H3K27me3) to repress gene expression in Arabidopsis (Shirzadi et al., 2011). However, demethylated DNA regions are a prerequisite for H3K27me3 deposition, hence PcG proteins-mediated transcriptional gene silencing should be constructed as secondary regulatory mechanisms of imprinting (Jiang and Kohler, 2012; Batista and Köhler, 2020). PcG proteins are either recruited to genes with a specific methylation pattern or work in conjunction with histone modifications, particularly adding a repressive mark called H3K27me3 to certain genes. The addition of H3K27me3 by PRC2 is recognized by Polycomb Repressive Complex 1 (PRC1), which further compacts the chromatin, reinforcing the silencing of the target gene. This multistep process ensures robust and heritable gene repression during development (Gehring et al., 2011; Rodrigues and Zilberman, 2015). Studies also indicated that there can be dual epigenetic regulation in seed development. For instance, AGL36 parent-of-origin-dependent expression is regulated by METHYLTRANSFERASE1 (MET1), DEMETER (DME) DNA glycosylase, and the components of the FIS-PRC2 complex. Among the regulatory players, lncRNAs are known to interact with PRC2 complexes, in both plants and animals. For instance, lncRNAs like HOTAIR (in animals) recruit PRC2 to specific genomic sites to induce histone methylation modifications [51]. In plants, the evidence for lncRNAs recruiting PRC is still emerging. A study highlights that cold response lncRNA COLDAIR in Arabidopsis has been shown to interact with the PRC2 component CURLY LEAF (CLF), leading to the deposition of the H3K27me3 mark and subsequent transcriptional repression at the FLOWERING LOCUS C (FLC) during vernalization (Jiang et al., 2008; Shen et al., 2021). This suggests that plant imprinting machinery might be similar to that of animals. However, further study involving the identification of ICEs and functional characterization of imprinted lncRNAs reported in model plants might reveal the potential role of lncRNAs in plant gene imprinting (Jiang et al., 2008; Zhang et al., 2011). It is likely that a more intricate interplay with other factors dictates the silencing patterns of imprinted genes.

## 3 From meiosis to maturation: the imprinting journey in seed development

Seeds are multi-generational structures containing a small embryonic plant enclosed in layers of diverse parental origins (Pires, 2014). Following meiotic events, several critical stages characterize the establishment and progression of imprinting during seed development. These include fertilization, embryogenesis, endosperm development, seed maturation, and germination (Baskin and Baskin, 2019). In fact, *de novo* DNA methylation during gametogenesis sets the imprints even before fertilization (Autran et al., 2005) implying that the maternal and paternal parents have different epigenetic contributions to seed development (Bai et al., 2016). A previous study in Arabidopsis suggests that maternal and paternal genomes have distinct roles in regulation seed size (Xiao et al., 2006). Post-fertilization events maintain the imprints and determine the monoallelic expression crucial for proper endosperm development and embryogenesis and other seed structures including seed coat development (Bauer and Fischer, 2011). In fertilization, a diploid zygote is produced by combining the genetic and epigenetic imprints from both parents. Disruptions in imprinted gene expression due to ploidy level changes can lead to abnormal seed development. The balance of maternal and paternal gene expression, achieved through parent-of-origin expression of imprinted genes, is crucial for regulating seed size and development in plants. This concept is sometimes referred to as dosage compensation. Therefore, differences in parental ploidy levels expected to alter the expression of imprinted genes which ultimately changes the seed phenotypes. An imbalance in genome dosage can lead to abnormal endosperm development, resulting in either excessive proliferation (larger seeds) or premature cellularization (smaller seeds). For instance, expression of a PEG gene identified to be PHERES1 (PHE1) increased in a paternal excess crosses, producing larger seeds possibly through extended proliferation of the endosperm (Batista et al., 2019). Similarly, MEG gene MEA (MEDEA) involved in suppression of endosperm proliferation causes early cellularization of endosperm and smaller seeds in a maternal excess crosses (Kinoshita et al., 1999; Kang et al., 2008). Disruption of FIE1 (MEG gene) balance through ploidy changes can lead to abnormal seed development (Ohad et al., 1996; Gutierrez-Marcos et al., 2003).

Estimating the imprinting/parent-of-origin effects on F1 can be challenging, as it is a multifactor-dependent trait in plants. Cytoplasmic organelles are typically inherited from the female, favoring the maternal role in F1 seeds (Bai et al., 2016). For instance, parent-of-origin expression patterns in the sorghum hybrid endosperm identified the maternal genotype as an effector in hybrid vigor, with most genes showing allele-specific expression being MEGs due to contributions from chloroplasts and mitochondria (Zhang et al., 2016b). Also, in conventional reciprocal crosses, maternal effects are significant, as imprinted gene expression in both nuclear and cytoplasmic genomes are confounded (Ashe et al., 2021). Hence, differentiating between genomic imprinting and maternal effects requires a combination of genetic, epigenetic, and environmental analyses. Reciprocal crosses are a common starting point, but further detailed investigations into allele-specific expression and epigenetic marks for imprinting, and cytoplasmic inheritance and maternal provisioning for maternal effects, are necessary to accurately distinguish between the two phenomena. Taken together, it is clear that the combined effects of maternal tissue contribution, environmental effects, resource provisioning, hormonal control, and dispersal and phenology of mother plants are some of the other factors could influence seed development (Baskin and Baskin, 2019). It is worth noting that the concept of “the imprint” in seed development is a complex area of research, and the specific events influencing the imprint may vary depending on the plant species. This chapter has explored the complexities of imprinting effects on endosperm and embryo development. Some of imprinted genes associated with seed development in flowering plants are listed in **Table 1**.

**Table 1:** A list of imprinted genes known for their roles in seed development across various plant species.

Gene Name	Parent of origin	Function	Species	Phenotype	Reference
Fertilization Independent Endosperm (*FIE*)- *OsFIE1* and *OsFIE2*	Maternal	Seed development	Rice	*osfie1* mutant lines produced smaller seeds and displayed reduced dormancy while *osfie2* exhibited impaired cellularization of the endosperm	(Cheng et al., 2020)
MEDEA (*MEA*)	Maternal	Seed development	Arabidopsis	Disruption of *MEA* gene leads to delayed development and over-proliferation of embryo and endosperm	(Kiyosue et al., 1999)
*MADS78* and *MADS79*	–	Regulate early seed developmental transition	Rice	Seeds overexpressing *MADS78* and *MADS 79* exhibited delayed endosperm cellularization, while CRISPR-Cas9-mediated single knockout mutants showed precocious endosperm cellularization.	(Paul et al., 2020)
Fertilization Independent Seed 2	Maternal	–	Arabidopsis	The *fis1* and *fis2* mutants exhibit autonomous endosperm development, but the seeds remain partially developed and ultimately atrophy, with embryos failing to advance beyond the globular stage in the absence of fertilization	(Chaudhury et al., 1997)
AGAMOUS-LIKE 62 (*AGL62*)	Maternal	–	*Arabidopsis*	AGL62 is necessary to establish early seed development. *AGL62* is crucial in coordinating endosperm and seed coat development and in determining the timing of endosperm cellularization	(Figueiredo et al., 2015)
Decrease in DNA Methylation 1 (*DDM1*)	Maternal	–	Arabidopsis	*ddm1* mutants showed impaired heterosis and increased expression of non-additively expressed genes related to salicylic acid metabolism	(Zhang et al., 2016a)
SHORT HYPOCOTYL UNDER BLUE1 (*SHB1*) and *IKU2*	–	Seed development	Canola and Arabidopsis	Over accumulation of IKU2 and SHB1 increases seed mass.	(Xiao et al., 2016)
TRANSPARENT TESTA GLABRA 2 (*TTG2*)	Maternal	Seed coat pigmentation	Arabidopsis	Production of mucilage and tannin in seed coats	(Johnson et al., 2002)
MINISEED3 (*MINI3*) and ANGUSTIFOLIA3 (*AN3*)	Maternal	Regulate seed mass	Arabidopsis	Loss-of-function mutant, *an3-4*, exhibit increased seed mass. Seed embryo development is modulated via an AN3-MINI3 gene cascade	(Meng et al., 2016)
DNA methyltransferase MET1	Maternal	Maintains methylation of symmetric CpG	Arabidopsis	Initiate endosperm development in the absence of fertilization in *mea-1*/*MEA*	(Schmidt et al., 2013)
NUCLEAR RNA POLYMERASE D1 (*NRPD1*)	Maternal	Balances maternal and paternal genomes in the endosperm	*Arabidopsis*	Loss of the **NRPD1** gene in the paternal parent prevent seed abortion	(Satyaki and Gehring, 2019)
HOMEODOMAIN GLABROUS 9 (*HDG9*)	Maternal		Arabidopsis	Gene knock-out increase seed size	(Cao, 2024)
EARLY FLOWERING IN SHORT DAYS (*EFS*)	Maternal, Paternal	Seed size	Arabidopsis	*efs* mutant produces larger embryo that results in enlarged seeds	(Cheng et al., 2018)
At5g24240 (*MOP9.5*)	Maternal	–	Arabidopsis	MOP9.5 expression was reduced in *efs* mutants.	(Cheng et al., 2018)
*DELLA*	Maternal	key repressors of gibberellin responses and controls seed size	Arabidopsis	The gain-of-function *della* mutant (*gai-1*) produces larger seeds. Also DELLA activity activate the expression of *AINTEGUMENTA*, a genetic factor to control cell proliferation and organ growth in the ovule integuments	(Gomez et al., 2023)
PHERES1 (*PHE1*)	Paternal	Transcriptional regulator of imprinted genes	Arabidopsis	A transposon-induced disruption of *PHE1* significantly improved fertility. Antisense suppression of *PHE1* partially rescues the seed death caused by a loss of *mea* function	(Josefsson et al., 2006)
Nuclear RNA Polymerase D1 (*NRPD1*)	Maternal	*NRPD1* represses maternal genome dosage	Arabidopsis	In crosses between diploid females and tetraploid males, tetraploid *nrpd1* mutant fathers repress seed abortion, whereas diploid *nrpd1* mothers have no effect.	(Satyaki and Gehring, 2019)
MULTICOPY SUPPRESSOR OF IRA 1 (*MSI1*)	Paternal	Proper initiation and progression of seed development.	Arabidopsis	Seed abortion ratio (of 50%) was noted in heterozygous *msi1* when the mutant allele is maternally inherited. *msi1* gametophytes initiate endosperm development in the absence of fertilization	(Köhler et al., 2003)
***ZmYuc1*/**YUCCA1	–	Auxin biosynthesis	maize	Impaired IAA biosynthesis and defective endosperm in mutants	(Bernardi et al., 2012)
AGAMOUS-LIKE 36 (*AGL36*)	Maternal	–	Arabidopsis	No obvious difference in endosperm development between mutant and wild types	(Shirzadi et al., 2011)
ATHILA TRANSPOSONS	Paternal	–	Arabidopsis	The expression of ATHILA retrotransposons induce sterility in cross-species hybridizations	(Keith Slotkin, 2010)
Zm00001d030305 (*Zm305*)	Maternal	Kernel development	Maize	The immature and mature kernel areas were significantly larger in overexpression lines whereas the length and width of immature and mature kernels in the two knockout lines decreased significantly	(Dong et al., 2023)
ZHOUPI (*ZOU*)	–	–	Arabidopsis	Particiapte in lysis of the transient endosperm and formation of embryo cuticle	(Yang et al., 2008)
TRANSPARENT TESTA 8 (*TT8*)	Maternal	triploid block	Arabidopsis	*TT8* is essential for regulating paternal genome dosage, as loss of function in *tt8* leads to a complete rescue of the triploid block, allowing normal seed development.	(Zumajo-Cardona et al., 2023)
*MEA, FIS2, FIE* and *DME*	Maternal	Seed development	–	*mea*, *fis2*, and *fie*, and *dme* mutant allele from the female causes endosperm overproliferation, embryo arrest and seed abortion	(Gehring et al., 2004)
Os07g20110 (*MEG2*), Os06g30280 (*MEG3*)	Maternal	Seed development	Rice	Seed length, width and thickness, starch content, seed weight, embryo size were reduced in (*meg2-1* and *meg2-2*) mutants. *meg3* mutant, the 1000-seed weight and thickness of the seeds were reduced.	(Yuan et al., 2017)
Os01g08570 (*PEG1*), Os10g04980 (*PEG2/OsFBX365*) and Os10g37540 (*PEG3*/*OsFBDUF48*)	Paternal	Seed development	Rice	Defective *peg1* causes small and empty seeds. Seed weight reduced in *peg2* mutant. *PEG3* (*OsFBDUF48*), encoding a peg3 produces small seeds and reduces grain yield.	(Yuan et al., 2017)

In **Table 1**, the lack of clarity or unavailability of information is denoted by a hyphen.

### 3.1 Endosperm development

Imprinting gene expression or parent-of-origin effects are most profound in the endosperm of developing seeds, since endosperm is subject to conflicting parental interests over offspring provisioning (Povilus et al., 2018; Picard et al., 2021). It is a genetically biparental product of a double fertilization process and its development comprises a series of transitions controlled by both genetic and epigenetic mechanisms initiated after double fertilization (Tonosaki et al., 2021). Endosperm is essential for nourishing the developing embryo and is critical for seed dormancy and germination. It also acts as a reproductive barrier between distinct species and individuals with different ploidy levels, leading to speciation events in flowering plants (Köhler et al., 2021). Specifically, endosperm prevents the hybridization of newly formed polyploids with their non-polyploid progenitors, a phenomenon known as the triploid block. Endosperm development is regulated by a complex interplay of pathways involving type I MADS-box transcription factors, auxin and abscisic acid signaling pathways, and various epigenetic regulators (Bente and Köhler, 2024). Studies have demonstrated that both the timing and efficiency of the cellularization process play significant roles in determining seed size (Zhou et al., 2021). Also, the transition from the coenocytic stage (a multinucleate state without cell walls) to the cellular stage is a critical phase in endosperm development. The transition to the cellular stage involves the formation of cell walls around each nucleus, compartmentalizing the endosperm into individual cells. In Arabidopsis, studies have shown that mutations affecting endosperm cellularization lead to embryo arrest and seed abortion, indicating the essential role of this process in embryo viability (Baroux et al., 2007). Molecular genetic studies on early endosperm development have revealed an intricate interaction between parental genomes among others (Zhou et al., 2021). In particular, MADS-box protein AGL62, together with the paternally expressed imprinted gene PHERES1, controls cellularization in endosperm development (Tonosaki et al., 2021). It is also worth noting that parental genome balance in interspecific or interploidy crosses can alter the timing of cellularization (Sekine et al., 2013). Mutation in the maternal EMBRYONIC FLOWER2a (OsEMF2a), encoding a zinc-finger component of PRC2, causes nuclear divisions in the central cell even in the absence of fertilization and delayed developmental transitions in the endosperm after fertilization resulting in seed abortion (Tonosaki et al., 2021). However, seed abortion can be rescued by a wild-type paternal allele, suggesting strong reciprocal interaction between maternal and paternal genomes in seed development.

Strong lines of evidence proved that genetic composition of the endosperm and the parental imprinting are crucial for establishing a dynamic balance between maternal and paternal gene dosage in the endosperm. Maintaining a correct balance (2:1 maternal to paternal genomes) is essential for proper endosperm development (Johnston et al., 1980). Crosses between individuals of different ploidies result in an imbalanced maternal to paternal ratio of chromosomes in the endosperm. This imbalance often disrupts endosperm development and function leading to seed abortion or stunted growth (Pires, 2014). Research indicates that a paternal excess genome accelerates the rate of mitotic division and delays cellularization of endosperm, often leading to larger and heavier seeds (Pires, 2014). Conversely, excess maternal contribution results in reduced mitotic divisions and precocious cellularization, leading to seed abortion or smaller, lighter seeds (Pires, 2014), indicating differential impacts on final seed size and viability depending on parent of origin. One likely explanation is that greater paternal control over endosperm development draws more resources to offspring, while greater maternal control attempts to allocate resources more uniformly across all seeds (Povilus et al., 2018). The molecular dissection of endosperm development over parental genome dosage showed that the timing of endosperm cellularization is particularly sensitive to the balance of parental genomes (Pires, 2014). The duration of the syncytial phase of endosperm is positively correlated with endosperm and seed size (Olsen, 2020). Inappropriate ploidy levels or gene dosage in the endosperm often led to post-zygotic reproductive isolation, suggesting that the ploidy level of the endosperm is central to seed viability (Pires, 2014; Ashe et al., 2021). Also, CpG methylation has been shown to restrict cell proliferation in the sporophytic integuments, the tissues that surround and protect the developing seed and suppress paternal genes for ensuring proper endosperm development (FitzGerald et al., 2008). Paternal hypomethylated genome causes early endosperm cellularization, while hypomethylated maternal genome associated with delayed endosperm development in F1 seeds (Scott et al., 1998).

Molecular studies have identified expression patterns of genes such *CYCB1;1*, OsFIE1, Short Hypocotyl Under Blue 1 (*SHB1*), Agamous-like 62 gene (*AGL62*), and putative H3K27 methyl transferase that are directly or indirectly associated with endosperm cellularization (Olsen, 2020). However, only a fraction of the genes and regulatory elements involved in endosperm-based hybridization barriers have been identified. While these genes are associated with endosperm cellularization, further research is needed to determine if a common genetic pathway underlies their role in reproductive isolation. Nonetheless, genome imprinting, which results in monoallelic expression, affects the timing of endosperm cellularization depending on its parent of origin. For instance, plant-specific RNA polymerase IV (Pol IV), required for methylation-associated siRNAs, has antagonistic impacts on gene expression in parents, thus controlling the endosperm gene expression (Satyaki and Gehring, 2022). Endosperm and its development play a critical role in establishing and regulating an ecologically important adaptive trait in plants known as seed dormancy (Piskurewicz et al., 2016). Investigation of genomic imprinting in the mature seed endosperm has identified several imprinted genes participating in seed dormancy (Piskurewicz et al., 2016). In particular, the ABA produced and released to embryo by the endosperm of dormant seeds is the predominant factor modulating embryo growth and seed dormancy (Lee et al., 2010). Molecular data indicate that upon imbibition, ABA stimulates the expression of LATE EMBRYONIC ABUNDANT (*LEA*) genes, which inhibit embryonic lipid catabolism (Penfield et al., 2006). Seed dormancy levels were maternally controlled or influenced through maternal inheritance (Piskurewicz et al., 2016). In another study, the H3K27me3-regulated *MISSEN* lncRNA was found to suppress nucleus division, distribution, and endosperm cellularization by blocking the function of HeFP, impairing cytoskeletal polymerization during endosperm development (Zhou et al., 2021).

### 3.2 Embryo development

While most molecular studies on imprinting focus on endosperm development and agronomic traits such as seed number and size, there is a significant gap in research investigating the contribution of imprinted genes to embryo development (Raissig et al., 2013). Unlike ephemeral tissue, endosperm, imprints of embryo are erased during gametogenesis. This event particularly helps plants to achieve parent-of-origin specific gene expression in the embryo. For instance, the maize imprinted gene maternally expressed in embryo 1 (*mee1*) maintains its methylation marks in the endosperm, while the embryonic maternal allele is demethylated upon fertilization and remethylated later in embryogenesis (Jahnke and Scholten, 2009). Genomic imprinting, resulting in parent-of-origin-dependent gene expression is crucial for balanced influence of maternal and paternal genetics and embryo viability (Pires et al., 2016). In Arabidopsis, the paternal genome is crucial for effective endosperm and seed development (Shirzadi et al., 2011). In its absence, several hundred genes, including AGAMOUS-LIKE (*AGL*) genes encoding Type-I MADS-box transcription factors, are downregulated (Shirzadi et al., 2011). Maternally derived *AtLETM2* is essential for seed development; its absence in an *Atletm1* mutant background results in early seed abortion (Zhang et al., 2012). A study focusing on cell-lineage-specific and allele-specific transcriptome revealed that paternal and maternal genomes contribute equally to the transcriptomes of both the apical cell lineage and the basal cell lineage of early proembryos (Zhao et al., 2020). However, a strong maternal effect on basal cell lineage development reveals differences between transcriptome and the phenotype. This study indicates that equal parental contribution to the transcriptome is not necessarily coupled with equivalent parental control of proembryonic development. Interestingly, the comparative transcriptome analysis between ACL and BCL shows more parent-of-origin genes in the BCL (Zhao et al., 2020). Moreover, maternally derived dry pericarp and its chemical constituents in the soil were shown to contribute to seed longevity by deterring microbial degradation, enhancing seed germination, and improving seedling establishment and vigor thus suggesting maternal dominance in seed development (Godwin et al., 2017).

Imprinted genes participate in zygote polarization, a critical initial step in early embryogenesis (Wang et al., 2021). Genes such as MAPKK kinase (*MAP3K*) YODA (*YDA*), BRASINOSTEROID SIGNALING KINASE 1 (*BSK1*), *BSK2*, EMBRYO SURROUNDING FACTOR 1 (*ESF1*), SHORT SUSPENSOR (*SSP*), and receptor kinase ERECTA show differential impact on zygote polarization and embryo development through parent-of-origin dependent gene expression (Wang et al., 2021). The BABY BOOM (*BBM*) AINTEGUMENTA-LIKE (*AIL*) AP2/ERF domain transcription factor is imprinted in both endosperm and embryo was shown critical for the maintenance of zygotic embryo development (Chen et al., 2022). Parent-of-origin genes display developmental-stage-dependent and cell-lineage-dependent allelic expression patterns (Zhao et al., 2020).

## 4 Unlocking hybrid vigor: the power of parental coordination in seed development

Hybridization is widely used in crop breeding to harness hybrid vigor effects and generate improved phenotypes, forming the basis of modern agriculture (Castillo-Bravo et al., 2022). However, gene imprinting adds another layer of complexity, impacting both hybrid vigor and seed failure. Disrupted imprinting patterns can sometimes enhance hybrid vigor but more often lead to seed failure. Therefore, understanding the molecular basis of incompatible hybridization in intra- or interspecific crosses provides insight into reproductive barriers. Hybrid plants with mismatched maternal and paternal alleles exhibit reproductive isolation (Köhler and Weinhofer-Molisch, 2010). These differences are attributable to parental genomes evolving to varying degrees due to natural selection or genetic drift. Therefore, the strength of reproductive isolation can differ within or between the species. In most interploidy crosses, hybrid seed inviability results from differences in the genetic and epigenetic landscapes of the endosperm, known as the triploid block. Early-onset hybrid inviability due to parental conflict is a powerful intrinsic reproductive barrier in seed plants (Coughlan et al., 2020) might be useful in preventing gene pool mixing and maintaining distinct identities. Hybrid seed inviability (HSI) is rapidly evolving, with the populations exhibiting the highest levels of HSI being the closest relatives to each other. Hybrid seed defects often follow a parent-of-origin pattern, suggesting that differences in number or expression strength of parent-of-origin-specific imprinted genes are primary or the secondary causes of the endosperm balance number (EBN) (Lafon-Placette et al., 2018). For instance, methylation status of the promoter of Circadian Clock 55 Associated1 (*CCA1*) controls imprinted gene expression, impacting hybrid vigor (Ng et al., 2014). F1 hybrid seed lethality is common in crosses between closely related diploid species (Garner et al., 2016), posing a significant obstacle to plant breeding. Research aims to understand how imprinting influences seed development and how to manipulate it for breeding purposes.

Crossing studies including Arabidopsis interploidy crosses show that endosperm failure, rather than intrinsic F1 hybrid incompatibilities, is the major cause of embryo and seed abortion (Städler et al., 2021). Improper dosage of normally imprinted genes in the endosperm lead to irregular growth and inviability possibly due to whole-genome duplication, gene variants, and duplication in intra-diploid crosses, and ploidy dosage in Interploidy crosses (Coughlan et al., 2020). Asymmetries in seed size and developmental trajectories reflect parental divergence in dosage-sensitive processes. Hence, endosperm balance number (EBN) also known as ‘effective ploidy’ can be used to quantify conflict strength between species (Lafon-Placette et al., 2018; Liu et al., 2020). Paternally expressed genes (PEGs) are associated with transposable elements and their silencing mark, DNA methylation, suggesting that transposable elements drive imprintome divergence between species (Lafon-Placette et al., 2018). Reports suggest that the paternally derived genome significantly influences F1 seed size, with genetic hybridity enhancing genome dosage effects, thereby enhancing heterosis in plants (Castillo-Bravo et al., 2022).

Investigating parental ploidy influence on seed development in oil/food crops is essential to understanding hybrid vigor. Literature shows that the maternal parent influences various aspects of seed development, including the seed size and shape, by transferring the genetic material to the seed, such as cytoplasmic factors and genes located on the sex chromosomes (Marcel et al., 2024). In contrast, paternal cytoplasm plays a major role in the post-plasmogamic events such as cell wall formation, gamete nuclear migration and fusion, and zygotic cell elongation and asymmetric division in zygote (Ohnishi and Kawashima, 2020). Maternal effects on seed size are attributable to the inheritance of cytoplasmic organelles, gametophytic effects, sporophytic effects, and genomic Imprinting (Castillo-Bravo et al., 2022). Likewise, paternal effects can occur through male gametogenesis or post-fertilization (Batista and Köhler, 2020). Studies indicate that the dosage of parental genomes is crucial in determining parent-of-origin effects on seed endosperm development and seed size. An excessive paternal genome can result in prolonged endosperm proliferation and abnormally large seeds, while an excessive maternal genome can cause precocious endosperm cellularization, leading to small F1 seeds (Castillo-Bravo et al., 2022). However, triploid block due to lethal disruption of endosperm development was restricted to paternal excess, with maternal excess crosses yielding viable seed in *Brassica oleracea* suggesting parent-of-origin effects on seed development can vary to plant species or genes (Stoute et al., 2012). Therefore, parental genome dosage effects can be effectively utilized to enhance genetic hybridity effects in F1 hybrids generated from inter-ploidy crosses.

The interplay between parental genome dosage and imprinting can be seen as a balance between conflict and cooperation. While both parents contribute genes promoting their own offspring success, imprinting and dosage regulation mechanisms ensure optimal seed development. In typical seed development, especially in flowering plants, the endosperm forms with a 2:1 maternal-to-paternal genome ratio. Deviations from this ratio can lead to improper regulation of imprinted genes. An excess of maternal genomes (e.g., 3m:1p) or paternal genomes (e.g., 1m:2p) disrupts the normal imprinting patterns, leading to developmental abnormalities (Scott et al., 1998; Erilova et al., 2009). When crosses occur between species or ploidy levels, the resulting seeds often exhibit an imbalanced genome dosage. This imbalance affects the expression of imprinted genes, often leading to a phenomenon known as the triploid block, where the seed fails to develop properly (Erilova et al., 2009). In cases of triploid block, the endosperm may not develop correctly due to the disrupted dosage balance, affecting nutrient allocation and leading to seed inviability. Imprinted genes are regulated through epigenetic modifications such as DNA methylation and histone modifications. The correct parental genome dosage ensures the proper establishment and maintenance of these epigenetic marks. The proper dosage ensures that the right alleles (either maternal or paternal) are expressed while the other is silenced. Disrupted dosage can lead to the loss of imprinting (LOI), where both alleles might be expressed or silenced inappropriately. Parental genome dosage plays a critical role in regulating gene imprinting, which in turn influences various developmental processes in plant seeds. Proper dosage ensures the correct expression of imprinted genes, leading to balanced growth, nutrient allocation, and seed viability. Disruptions in dosage can lead to developmental abnormalities, hybrid seed failure, and reproductive barriers. Understanding these relationships is crucial for advancing plant breeding and improving crop yields.

To conclude, the relationship between parental genome dosage and genome imprinting in plant seed development is complex, intertwined and is fundamental to seed development. Balanced genome dosage ensures that the seed develops properly, while imprinting regulates gene expression to optimize growth and resource allocation. Disruptions in either can lead to developmental issues, highlighting the importance of their coordinated action in successful seed development. By manipulating imprinting and genome dosage, plant breeders can potentially overcome barriers to hybridization and create new varieties with desirable traits.

## 5 The evolutionary significance of genomic imprinting

The evolutionary significance of genomic imprinting in plants lies in its role in mediating parent-offspring conflict (Pires, 2014), maintaining species boundaries (Erilova et al., 2009), facilitating adaptation (Ohto et al., 2005), and balancing genetic contributions (Erilova et al., 2009). It influences crucial developmental processes and traits, contributing to the evolutionary fitness and success of plant species (Rodrigues and Zilberman, 2015). The exact mechanisms by which imprinting evolved, manifests, and is maintained are still being explored. However, several hypotheses, including parental conflict theory (Scott et al., 1998), defense hypothesis (Yoder et al., 1997), and coadaptation (Wolf and Hager, 2006) explain the evolutionary origin and importance of genomic imprinting in plants (Köhler and Weinhofer-Molisch, 2010; Patten et al., 2014; Rodrigues and Zilberman, 2015). The parental conflict theory proposes that proposing that imprinting in endosperm arose as a consequence of an intragenomic conflict over the distribution of resources in the developing seed. Imprinting allows for maternally expressed genes as they might promote efficient resource use within the seed, ensuring the developing embryo gets what it needs while the paternally expressed genes might promote faster growth or larger seed size, potentially increasing seed dispersal or competitiveness (Ashe et al., 2021). This creates a balance between ensuring offspring survival (maternal interest) and maximizing seed production (paternal interest). The distinctive mechanisms of gene imprinting in the endosperm suggests that flowering plants might have coevolved double fertilization and imprinting to prevent parthenogenetic development of the endosperm (Huh et al., 2008). On the other hand, Defense hypothesis theory proposes that imprinting might be a side effect of a defense mechanism against transposons in gametes (Jiang and Kohler, 2012). Transposons can disrupt genes and harm the developing embryo. Therefore, methylation tags might be placed on certain genes to silence potentially harmful transposons during gametogenesis. These methylation tags might persist after fertilization, leading to imprinting of the gene depending on its parent of origin. In other words, defense hypothesis theory suggests imprinting is not directly related to resource allocation but could be a consequence of silencing harmful genetic elements. Both theories have some evidence supporting them, but the exact role of imprinting in plants might be a combination of both factors. Research suggests imprinted genes are often found in the endosperm supporting the resource allocation (Spencer and Clark, 2014).The sexual antagonism and maternal-offspring coadaptation theories proposes that genomic imprinting as a consequence of natural selection for superior performance of offspring. There is also a possibility that imprinting may have evolved to provide a mechanism for rapid neofunctionalization of genes during seed development to increase phenotypic diversity of seeds as suggested by Bai and Settles (Bai and Settles, 2015). This could be one reason for the maintenance of imprinted genes in mainly self-fertilizing species, where any extent of genetic conflict is predicted to be low.

## 6 Future directions

For understanding the epigenetic modifications associated with imprinting is complex, and the regulatory process governing imprinting remain poorly understood for many crops. The genetically chimeric nature of seed tissues and epigenetic natural variation are major bottlenecks in the imprinting studies in plants. Identifying key imprinted genes associating with desired agronomic traits is an active research area in this field. Also, methods to manipulate imprinting or selection for desired parental contributions during breeding are being explored. It is worth mentioning that imprinting could be combined with traditional breeding methods or gene editing for even more targeted crop improvement. Therefore, exploiting imprinting for crop improvement is a promising future direction in breeding. By understanding the imprinting patterns, breeders could select for seeds with the desired parental contribution, leading to crops with improved agronomic traits like improved germination rates, enhanced stress tolerance and optimized seed dormancy for better storage or planting strategies. Nonetheless, understanding the intricate mechanisms of imprinting in different plants requires further research and in some cases, altering imprinting patterns might have unforeseen effects on plant development, requiring careful evaluation. At this juncture, future researches should focus on development of robust techniques for easy and comprehensive identification of imprinted genes and their functional characterization as it is expected to deepen our understanding on genomic imprinting. Application of chromatin immunoprecipitation (ChIP), and DNA methylation profiling or the development of machine learning algorithms trained on ICEs of other species can be effective in identifying imprinting control elements, which is a key imprinted gene regulator. Moreover, comparing the imprinted genes and their functions across diverse plant species might reveal broader evolutionary patterns and their impact on development. This could aid in development of robust computational models to simulate the impact of imprinted gene disruptions and predict their consequences for plant fitness in new plant model system. Additionally, efforts should be made to combine molecular biology techniques with ecological and evolutionary studies to understand the real-world implications of imprinting. Also, the current literature suggests a stronger focus on imprinted genes in relation to seed endosperm development, particularly seed size and number, compared to their role in embryo development itself. This information suggests a clear need for further research on imprinted genes in embryo development. Therefore, future studies focusing identification and characterization of embryogenesis-related imprinted genes and their inheritance pattern across multiple generations is unavoidable to address the existing knowledge gap in the field of genomic imprinting. Application of modern genetic manipulation tools like transgenic overexpression and gene knockouts with embryo-imprinted genes/candidate genes might shed light on how imprinted genes contribute to the intricate dance of maternal and paternal genetic information during embryo development. This knowledge can ultimately be translated into improved crop varieties with enhanced yields, resilience, and adaptation to changing environments. Overall, exploiting imprinting offers a novel approach for developing new crop varieties with improved traits or even treatments for certain seed developmental problems. As research progresses, it has the potential to revolutionize how we breed plants for a more sustainable and productive future.
